# Comparative Analysis of Strategies for *De Novo* Transcriptome Assembly in Prokaryotes: *Streptomyces clavuligerus* as a Case Study

**DOI:** 10.3390/ht8040020

**Published:** 2019-11-30

**Authors:** Carlos Caicedo-Montoya, Laura Pinilla, León F. Toro, Jeferyd Yepes-García, Rigoberto Ríos-Estepa

**Affiliations:** Grupo de Bioprocesos, Departamento de Ingeniería Química, Universidad de Antioquia UdeA, Calle 70 No. 52-21, Medellín 050010, Colombialaura.pinilla@udea.edu.co (L.P.); lfelipe.toro@udea.edu.co (L.F.T.);

**Keywords:** next-generation sequencing, Rockhopper2, *Streptomyces clavuligerus*, Transcriptome Assembly Quality Assessment, Trinity

## Abstract

The performance of software tools for de novo transcriptome assembly greatly depends on the selection of software parameters. Up to now, the development of de novo transcriptome assembly for prokaryotes has not been as remarkable as that for eukaryotes. In this contribution, Rockhopper2 was used to perform a comparative transcriptome analysis of *Streptomyces clavuligerus* exposed to diverse environmental conditions. The study focused on assessing the incidence of software parameters on software performance for the identification of differentially expressed genes as a final goal. For this, a statistical optimization was performed using the Transrate Assembly Score (TAS). TAS was also used for evaluating the software performance and for comparing it with related tools, e.g., Trinity. Transcriptome redundancy and completeness were also considered for this analysis. Rockhopper2 and Trinity reached a TAS value of 0.55092 and 0.58337, respectively. Trinity assembles transcriptomes with high redundancy, with 55.6% of transcripts having some duplicates. Additionally, we observed that the total number of differentially expressed genes (DEG) and their annotation greatly depends on the method used for removing redundancy and the tools used for transcript quantification. To our knowledge, this is the first work aimed at assessing de novo assembly software for prokaryotic organisms.

## 1. Introduction

The study of transcriptomes is essential for interpreting the genome functional elements and revealing their molecular constituents [1]. Goals for studying transcriptomes include identifying transcripts, characterizing transcript structural complexity and coding content, and more important, understanding which genes are expressed in different samples under various conditions and their levels of expression [2,3].

Traditionally, microarray analysis has been the technique used for profiling the global expression of genes. This hybridization-based technology is restricted to known genes and has a limited range of quantification. Nowadays, RNA sequencing (RNA-seq) is the most popular procedure; it consists of an experimental methodology that uses next-generation sequencing (NGS) technologies to generate DNA sequence reads derived from the entire RNA molecule to determine the primary sequence and relative abundance of each RNA [2,4].

Once sequencing is performed, the resulting reads are aligned to either a reference genome or reference transcripts, or assembled de novo without the genomic sequence, thus acquiring a complete transcription map [1]. De novo transcriptome assembly is necessary for organisms whose genomes have been neither sequenced nor annotated, e.g., for non-model organisms, when analyzing complex microbial communities, in meta-transcriptome studies, or while investigating uncultivable microorganisms [5,6,7]. Many software tools have been developed to assemble transcriptomes using the de novo strategy. The most commonly used are: Trinity [3,8], Oases [6], Bridger [9], SOAPdenovo-Trans [10], IDBA-Trans [11], SSP [12], Shannon [13], BinPacker [14] and Rockhopper2 [5]. 

De novo assembly is very sensitive to software parameters due to the lack of a genome to guide the assembly and the type of algorithms used which are mostly based on the De Bruijn graphs. Thus, they depend on the k-mer length [14] or on the minimum k-mer coverage [3]. Moreover, the consistency and biological relevance of the data, obtained from different sources, make it challenging to select the most accurate assembly [15,16], and the same data can generate substantially different assemblies, both within and between assembly methods, affecting the biological analysis and its conclusions [17]. In this regard, some authors have undertaken the task of evaluating the impact of different methodologies and software configurations on the quality of the assembled transcriptome [18,19,20,21,22]. 

Different software are available for evaluating the quality of a de novo assembly, e.g., SCAN [23], rnaQUAST [24], DETONATE [16], Transrate [17] and recently, a topology-based method called Branching Measure [25]. For the case of Transrate, it renders a Transrate Assembly Score (TAS) that allows for not only selecting the best assembler but also optimizing and tuning the assembler’s parameters; for this, it uses solely the sequenced reads and the assembly as inputs [17]. In summary, assembly assessments are essential for the evaluation of new methods, or in the combination of assemblies as part of optimization strategies [26]. Since no package outperforms others, for the same dataset, very different assemblies are obtained depending on the parameters used. Hence, it is always a good practice to optimize the software parameters used for a given organism [15,19,27,28].

Furthermore, many assemblers and methodologies were developed using eukaryotic transcriptome data. Related works have not been performed in bacteria wherein processes, structures and transcriptional mechanisms are different, having transcriptional units that often overlap, polycistronic transcripts, different promoters that may drive expression of a gene or operon under different conditions, etc., [29,30]. At the time of writing this manuscript, only two software packages had been designed specifically for bacterial transcriptome: SPARTA [31] and Rockhopper2 [5]; both of them run on a reference-based mode but, for de novo transcriptome assembly, only Rockhopper2 has been implemented.

This work aimed at performing an assembler parameter optimization; having in mind that strategies for evaluating the quality of transcriptome assembly in prokaryotes are rather scarce, we assessed the incidence of software parameters on software performance for the identification of differentially expressed genes as a final goal. Further, we performed a comparison between assemblers as well as developed approaches to assess and remove redundancy during data processing. We found that the assembly method, the redundancy removal and transcript quantification affect the quantity and the identity of deferentially expressed genes, thus modifying the biological analysis and results.

## 2. Materials and Methods

### 2.1. RNA Extraction, Library Preparation and Sequencing 

*Streptomyces clavuligerus* (*S. clavuligerus*) American Type Culture Collection (ATCC) 27064 RNA samples were taken during the stationary phase of the cells, grown under favorable and unfavorable environmental conditions for clavulanic acid (CA) production. Experimental conditions were set up at 28 °C, pH 6.8 and shaking at 220 rpm. For the high CA production condition, an isolated soy-protein based medium was used [32]; for the low production condition, the chemically-defined media GSPG medium was employed [33]. RNA extraction was performed using the TRIzol® RNA Isolation Protocol [34]. The quantity of RNA was determined using a Nanodrop spectrophotometer. The RNA integrity number (RIN) was verified with the Agilent 2100 Bioanalyzer. For the cDNA library preparation, the TruSeq stranded mRNA Sample Preparation Kit (Illumina) was used. The Ilumina Hi-seq 2000 platform was utilized for RNA sequencing, and 2 x 101 bp paired-end libraries were obtained. These experiments were performed elsewhere [35] so the sequencing data was available for performing the current study. Raw RNA-seq data were deposited in the National Center for Biotechnology Information´s (NCBI) Sequence Read Archive (SRA) under accession number SAMN11046362. 

### 2.2. Pre-Processing of Raw Data

FastQC was used for checking the quality of raw data; [36]. Subsequently, SortMeRNA [37] was used to remove ribosomal RNA. The procedure continued with a filtering of sequences by quality and an adapter trimming process, using Trimmomatic [38]. The “paired-end data” and “Truseq3” options were selected. The trimming steps were ILLUMINACLIP and SLIDINGWINDOW, both of them using the default parameters. Finally, an error correction procedure was implemented with the package BBNorm [39]. The quality of the libraries was checked with FastQC after each step in the pre-processing of raw data to ensure a successful pre-treatment of reads.

### 2.3. Transcriptome Assembly 

Transcriptome assembly was performed using Rockhopper2 [5]. A parameter optimization was carried out through a factorial and two central composite experimental designs, developed using STATGRAPHICS Centurion XVI^®^. The software parameters were defined as variables; the response variable was the Transrate Assembly Score (TAS). Subsequently, the performance of Rockhopper2 was compared with that of Trinity [3,40], using TAS as response variable; Trinity was run using the default parameters.

### 2.4. Transcriptome Assembly Quality Evaluation

Additional metrics were also computed for analyzing the quality of the assembled transcriptomes. We evaluated the completeness of the transcriptomes based on evolutionary expectations of gene content from near-universal single-copy orthologues selected from the database OrthoDB by using the software BUSCO [41]. We built the representation of full-length reconstructed protein-coding genes, through BLASTN search by using the command ***analyze_blastPlus_topHit_coverage***, included in Trinity. Besides, we considered the percentage of fragments that mapped to the assembled transcriptomes, handed in by Transrate. Finally, the redundancy of transcriptomes was examined using BLAST homology search and the information in “outfmt6” format was processed using a Microsoft Excel spreadsheet. Moreover, analysis of vector contamination (VecScreen [42], and chimera detection (Vsearch [43] and CD-HIT-DUP [44]), both software implemented in the Galaxy platform [45,46], were utilized for detecting chimeras in the assembled transcriptome. The results allowed us to propose two methodologies for the elimination of redundancy. First, a software that cluster sequences based on sequence similarity was used; for this, we assessed CD-HIT [44]. Second, we removed redundancy based on a BLAST homology search, which was performed using TransPS [47].

### 2.5. Transcript Quantification and Differential Expression Analysis 

RNA-Seq by Expectation-Maximization (RSEM) [48] and Salmon [49] were used to evaluate the quantification of transcripts. Normalization and differential expression analysis was developed with the Bioconductor package EdgeR [50]. To be considered as differentially expressed (DE), genes must have a False Discovery Rate (FDR) <0.05. A gene with a fold-change larger than 2 (Log2FC >2) was considered upregulated. The dispersion parameter was set to 0.1 following the recommendations of the EdgeR manual for genetically identical organisms. A volcano plot, the application of a Bland-Altman plot (MA) plot and heat map were acquired following the scripts defined by Trinity [3]. Enrichment analysis for genes upregulated in both conditions (high and low production of CA) was performed through the R package GOseq [51]; for the visualization of the results we used REVIGO [52].

### 2.6. Annotation

Transdecoder, a companion of the de novo transcriptome assembler Trinity [3], was used to identify candidate coding regions within transcript sequences. Trinotate [53], powered by BLAST+ [54], HMMer [55], Signalp and TMHMM [56], was utilized for automatic functional annotation of transcriptomes. For all BLAST+ searches, a nucleotides database of twenty Streptomyces species was created, including *S. clavuligerus*. In addition, for BLASTX searches, all the terms associated with the Streptomyces genus were downloaded from the TrEMBL database. Finally, Trinotate uses the Swiss-Prot database to perform BLASTX searches and to obtain the gene ontology terms.

### 2.7. Referenced-Based Analysis 

**Figure 1:**
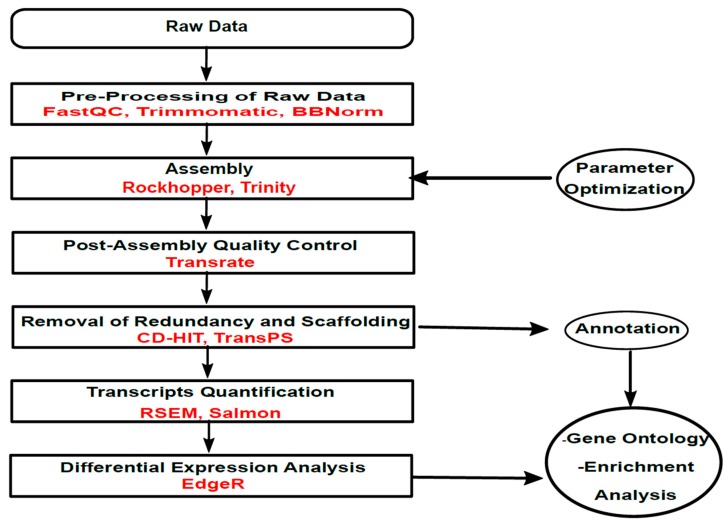
Workflow for the bioinformatics analysis implemented in this study.

A reference-based strategy was implemented in the Galaxy platform for comparing the results of the de novo strategy for transcriptome assembly. The reference genome and the annotations for *S. clavuligerus* were downloaded from Ensembl Bacteria [57]. Alignments to the reference genome was performed with Bowtie 2 [58]; for transcripts quantification, Htseq-count [59] was used. Normalization and differential expression analysis followed the same technique as for the de novo strategy. Figure 1 shows the corresponding workflow chart.

## 3. Results

### 3.1. Quality Control of Raw Data

Sequencing the libraries for the high and low CA production conditions produced high quality reads (Figure S1). The FastQC report shows an adapter contamination; the GC distribution over all sequences suggested a contamination with ribosomal RNA (sharp peak in the plot); the overrepresented sequences module confirmed this result. The GC content reached 57% for both libraries. Looking for possible contamination with rRNA, reads were aligned against the SILVA [60] and RFAM [61] databases, and the hits were removed with SortMeRNA [37]. Bases that showed suboptimal quality were trimmed, and the adapter content removed with Trimmomatic (Table S1 summarizes the results of the pre-assembly processes and the quality control). In addition, an error correction process, based on the k-mer content of the reads, was performed.

### 3.2. Transcriptome Assembly

**Figure 2:**
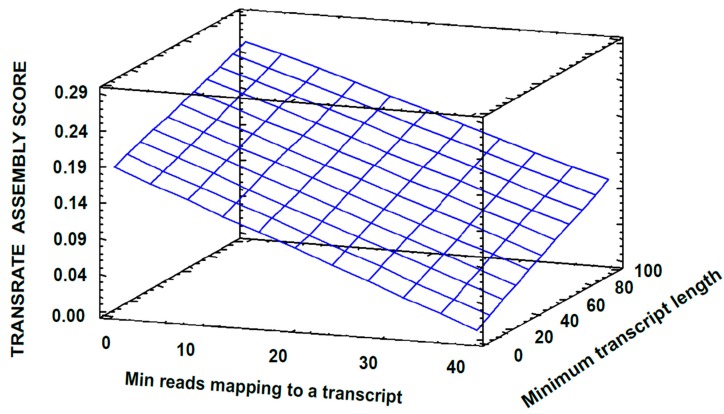
Response surface for the factorial experimental design aimed at evaluating Rockhopper2 parameters. *Min count to seed a transcript* = 50.0; *Min count to extent a transcript* = 5.0.

Rockhopper2 was initially used since it was developed specifically for bacterial transcriptomes [5]. Due to the fact that transcriptome assembly is a core stage of RNA-seq data analysis, and different assemblers produce different results for a given organism, it is always recommended to explore the parameter space to find optimized values [15,19]. Therefore, prior to any further use, the analysis began performing a parameter optimization for the software. First, a factorial experimental design was carried out (Table S2). Variables were defined as the Rockhopper2 parameters; Transrate Assembly Score (TAS) was set as the response variable. A TAS of 0.2746 was reached using the conditions set for the experiment 4 (Table S2), while for the default parameters, a TAS of 0.1331 was obtained. All parameters showed a meaningful impact on the value of TAS. The behavior of the response variable depending on the parameters value can be seen in Figure 2, wherein a TAS linear increment is observed with both an increase in “*B: Minimun transcript length”* and a decrease in “*A: Min reads mapping to a transcript”.*

**Figure 3:**
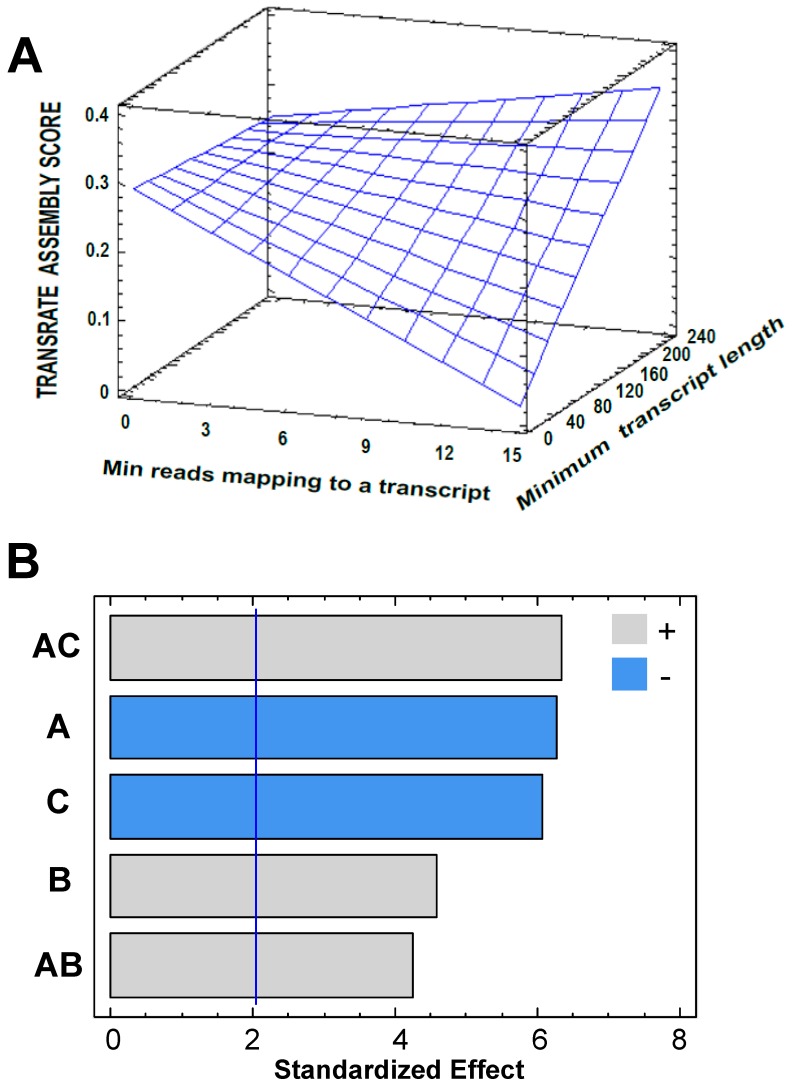
Central composite experimental design for the parameters of Rockhopper2, in the de novo mode. (**A**): Response surface. *Min count to seed a transcript* = 90.0; *Min count to extent a transcript* = 2.0. (**B**): Pareto chart for Transrate assembly score (TAS). A: *Min reads mapping to a transcript*; B: *Minimum transcript length*; C: *Min count to seed a transcript*. AC and AB are interaction between the previously defined factors

**Figure 4:**
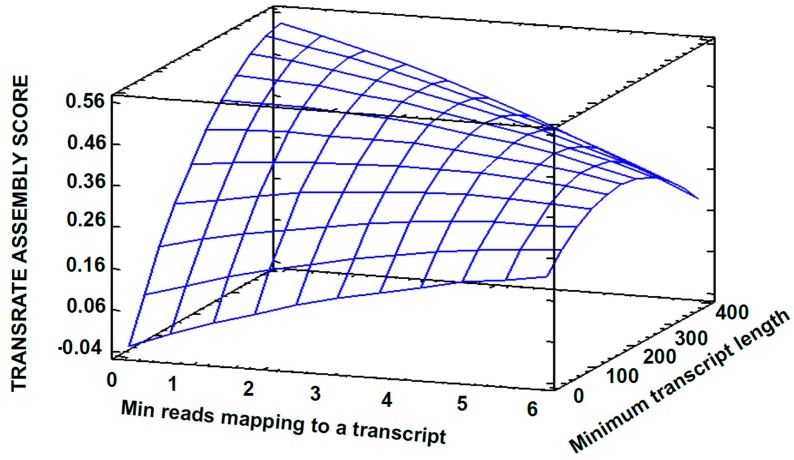
Estimated response surface for the Central Composite Experimental Design2. *Min count to seed a transcript* = 10.0.

Based on results from the factorial design, a central composite experimental design (CCD1) over the point with the maximum TAS (previously obtained) was proposed for continuing to improve the quality of the transcriptome assembly (Table S3). Process variables and the response variable were set as before. A TAS value of 0.3804 was reached with the conditions of Experiment 7; this is, A = 1 *(Min reads mapping to a transcript*), B = 160 *(Minimum transcript length*), C = 20 (*Min count to seed a transcript*) and D = 1 (*Min count to extend a transcript)* (Table S3). The parameter D did not show a significant effect on the TAS value (Figure 3); therefore, it was set to one and a new central composite design was established (CCD2). For this, the value of the parameter, when the maximum TAS was obtained, was set as the central point (Table S4); thus exploring values around this in an attempt for finding an optimum that contribute to a better assembly. Figure 4 shows the variation of the response variable as a function of the software parameters. The parameter B “*minimum transcript length*” caused the main effect on the response variable. The surface predicts a proportional TAS increase with this parameter. By default, Rockhopper2 uses 50 as a value for “*minimum transcripts length*”; this is a low value considering that the shorter the transcripts, the lower the probability of correspondence to real expressed transcripts.

Additional in silico experiments did not improve the TAS value, despite an increase in B: *minimum transcript length*. The optimum TAS value was 0.4885 (Run 17 in Table S4). A further improvement (TAS = 0.55092) was achieved by merging both libraries and assembling the transcriptome, an approach recommended by Trinity [3]. Table 1 shows the results from different metrics used for evaluating the quality of the transcriptome, assembled with Rockhopper2, both, and using the default and the optimized parameters. As observed, the quality of the assembled transcriptomes is very sensible to the software parameters:, thus, parameter optimization allowed us to reach a TAS of 0.55092; using the default parameters, the TAS was 0.19901 (merging both libraries). Additional measurements (such as the number of mapped fragments) particularly increased (75% using the default parameters and 88% using the optimized parameters). Error correction using BBnorm allowed a slight improvement in the transcriptome quality, thus rendering TAS values near 0.55709.

The transcriptome assembly was carried out in Trinity using the default parameters; the results were compared with Rockkhopper2 performance. A TAS of 0.58337 and 94% aligned fragments were observed. Table 1 shows the comparisons between the optimized Rockhopper2 and Trinity assemblers. Trinity outperforms Rockhopper2 despite the in-depth parameter optimization. According to Haas et al., Trinity reconstructs transcripts accurately with a simple and intuitive interface that requires either little or no parameter tuning [3].

Other metrics are improved by parameter optimization, e.g. “Number with Open Reading Frame (ORF)”, which indicates the transcripts that potentially encode for a protein. We calculated the mean length for the genes of the chromosome of *S. clavuligerus* ATCC 27064 as 343.06; the mean length calculated from the Trinity assembly is close to that from the optimized Rockhopper2.

### 3.3. Post-Assembly Quality Control

**Figure 5:**
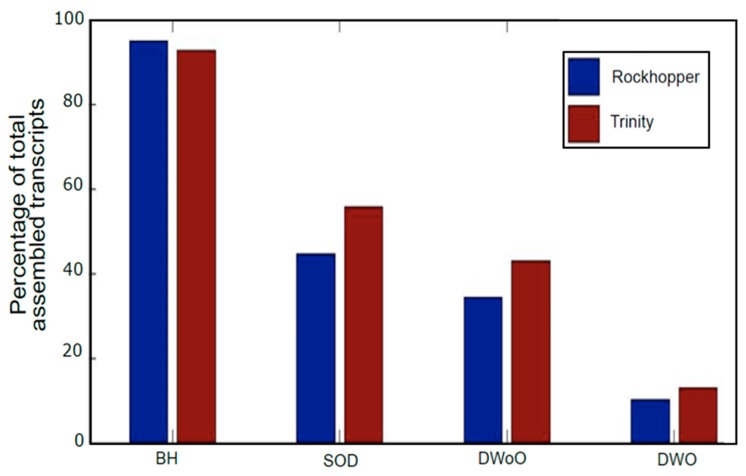
Post-assembly quality control. BLAST homology search. BH: BLAST Hits. SOD: Subjects with a least One Duplicate. DWoO: Duplicates WithOut Overlapping. DWO: Duplicates With Overlapping.

The analysis of the quality of the transcriptome cannot be limited to TAS, so further methodologies must be used, e.g., completeness assessment and redundancy determination. Two transcriptomes, obtained using different software solutions (optimized Rockhopper2 and Trinity), were exposed to different strategies to remove redundancy and completeness evaluation. First, redundancy was evaluated by searching for homologies in BLASTN (see Figure 5) [62]. The BLASTN search allows for identifying which transcripts that have the same hit are going to be scaffolded to a contig having a gap (duplicates without overlapping), or those that have superposition and therefore may be assembled to a larger contig (duplicates with overlapping). This redundancy of transcriptome library is inevitably existent since some transcripts, belonging to the same gene, have no overlapping regions so that they cannot be assembled together [63]. Moreover, searching homologies in BLASTN also permitted to ascertain additional errors obtained from Trinity, e.g., the same sequence assembled twice in the same transcript (self-chimeras). Though few cases were detected (6 out of 6488 assembled transcripts), this occurrence was not noticed in Rockhopper2.

In this regard, despite the fact that the TAS for Trinity surpasses that for Rockhopper2, assembly from Trinity shows larger redundancy, which needs to be removed. For this, two strategies were used: first, CD-HIT removed 115 transcripts with more than 80% identity with the transcriptome from Trinity. For the case of Rockhopper2, removal of redundancy using CD-HIT did not render results, which means that Rockhopper2 does not assemble contigs with at least 80% identity.

As a second strategy, we used scaffolding of contigs using TransPS. The methodology for TransPS deals with the search for homologies in BLASTX; based on such homology, scaffolding of contigs that codify for the same sequence is completed. In this manner, redundancy is removed, and, in some cases, longer transcripts are attained.

Consequently, three transcriptomes were achieved: Trinity.CD-HIT, Trinity.TransPS and Rockhopper.TransPS, with transcript numbers of 6373, 3285 and 2481, and TAS values of 0.6122, 0.5303 and 0.5405, respectively (see Table 2). The percentage of reads alignment to the assembled transcriptome was 94%, 83%, and 76%, respectively. For the removal of redundancy, TAS increases with CD-HIT and diminishes with TransPS.

**Figure 6:**
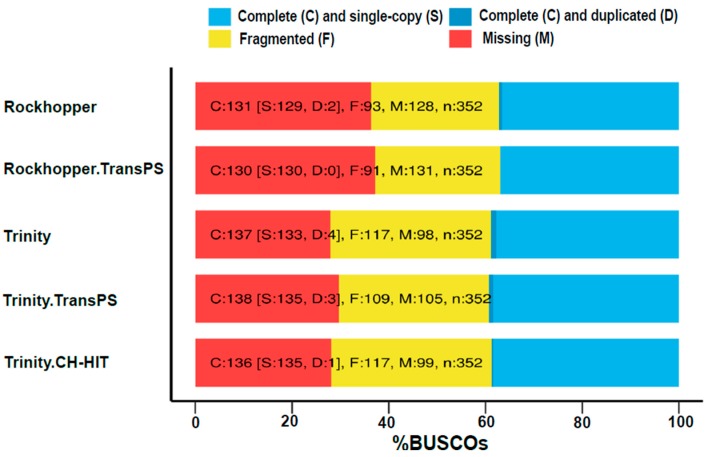
Benchmarking Universal Single-copy Orthologs (BUSCO) assessment results.

A further methodology used for testing transcriptome quality and completeness was Benchmarking Universal Single-copy Orthologs (BUSCO) (see Figure 6). As a summary, Trinity assembles transcriptomes with a larger number of fragmented transcripts, whereas Rockhopper2 presents a larger number of missing contigs. For both Trinity and Rockhopper2, the number of complete contigs was similar, though Trinity showed a tendency to cause more duplicates, which is consistent with results in Figure 5.

**Figure 7:**
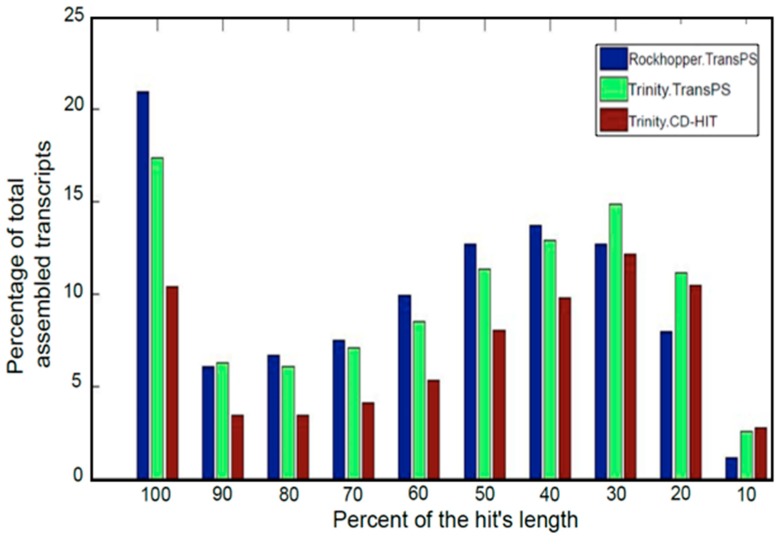
Full-length transcripts assembled retrieved from two assembly and two redundancy removal methods.

Finally, full-length transcripts were determined (see Figure 7). Ideally, all transcripts must have a percentage of the hit’s length equal to 100%, but due to the uneven coverage of the libraries, not all of the transcripts are assembled completely. Besides these metrics, an analysis of the assembled transcriptome was performed using VecScreen, for removing vector contamination. Nevertheless, for the case of the transcriptome assembled using Trinity, only 10 transcripts out of 6488 were detected with vector contamination, which represents only 0.15% of the total assembled transcripts. Also, Vsearch Chimera detection and CD-HIT-DUP were utilized in order to detect chimeras in the assembled transcriptome. No transcripts were categorized as chimera. Hence, an alternative methodology was proposed; it refers to aligning in BLASTN the transcripts with a score given by Transrate, sCseg less than 0.1, since a transcript with a low sCseg is possibly a chimera. Only three chimeras were found.

### 3.4. Transcript Quantification and Differential Expression Analysis

**Figure 8:**
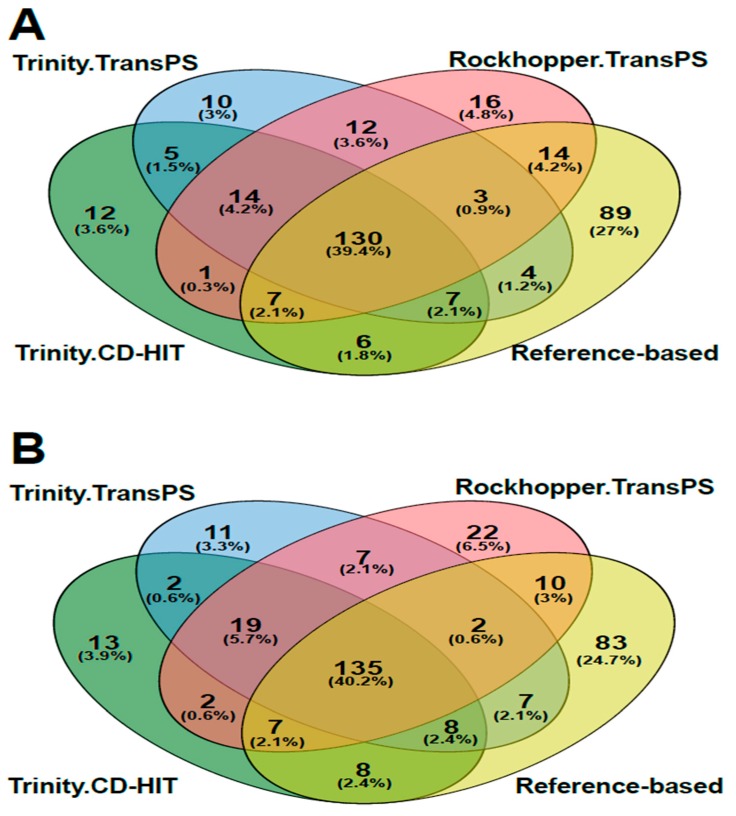
Differentially expressed gene analysis. (**A**): Venn diagram for differentially expressed genes (DEG) obtained using RNA-Seq by Expectation-Maximization (RSEM). (**B**): Venn diagram for DEG obtained using Salmon.

The resulting transcriptomes (Trinity.CD-HIT, Trinity.TransPS and Rockhopper.TransPS) were treated with the transcript quantification software RSEM and Salmon to compare an alignment-based method (RSEM) against an alignment-free method (Salmon). The first approach was to know how the software type for transcript quantification affects the number of genes classified as differentially expressed. Figure 8A,B show the differentially expressed genes (DEG) based on the software used. Results also include assembling outcomes using the *S. clavuligerus* genome as reference; from this, 97% of reads did align properly and 260 genes were detected as differentially expressed.

For the case of removal of redundancy using CD-HIT and quantification by both methods, we found transcripts with at least a duplicate in the group of DEG (25 and 26, using RSEM and Salmon, respectively). Once this software is used, problems related with redundancy are dragged all the way until the end of the DEG analysis. Results for the differential expression analysis for the Rockhopper.TransPS transcriptome are presented in the Supplementary Material (Figure S2A,B). As observed, a high proportion of genes detected as differentially expressed are upregulated in the high CA production condition. The gene differentially expressed with the greatest LogFC, i.e., the most upregulated, was a neutral-zinc metalloprotease, highly expressed jointly with a large group of proteases in the high CA production condition.

### 3.5. Annotation and Gene Ontology 

**Figure 9:**
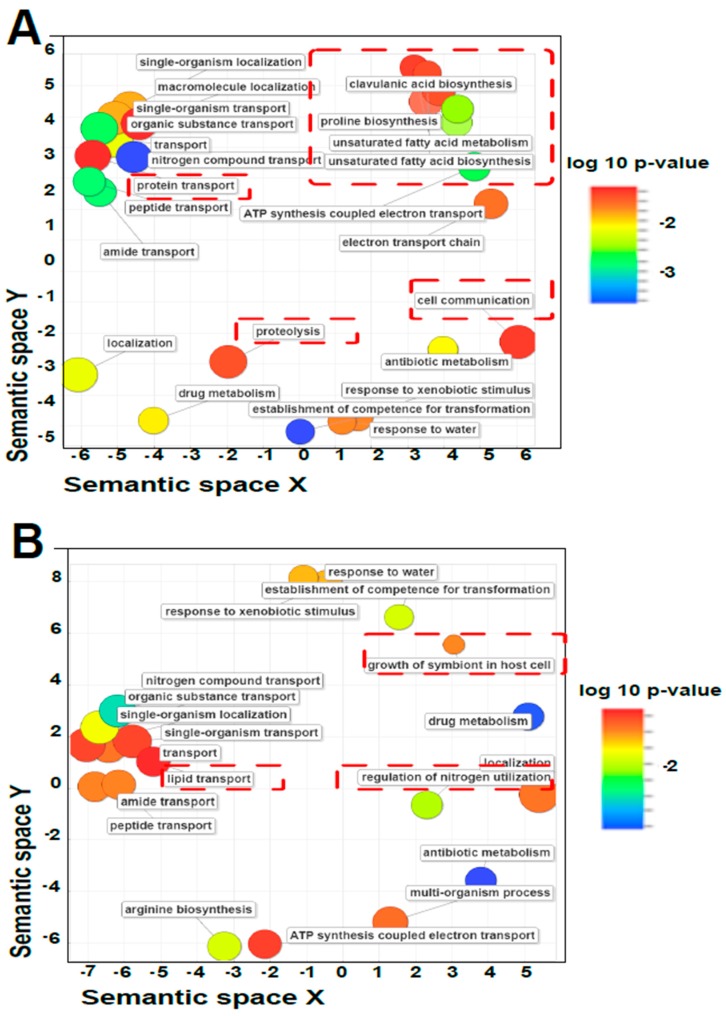
Gene ontology (GO) for biological processes (**A**): GO for upregulated genes related to clavulanic acid biosynthesis, using Trinity.TransPS (quantification method: Salmon). (**B**): GO for upregulated genes related with clavulanic acid biosynthesis, using Rockhopper.TransPS (quantification method: Salmon). Figures were obtained using the software REVIGO [52]. Highlighted with ----- are the nodes that differ from both annotations.

Figure 9 shows a summary for the GO results from the assembled transcriptomes. In Figure 9A, processes associated with CA biosynthesis, proline biosynthesis, cell communication, proteolysis and protein transport are enriched in this plot for the Trinity.TransPS transcriptome, while, for Figure 9B, regulation of nitrogen utilization, lipid transport and growth of symbiont in the host cell are enriched for the Rockhopper.TransPS transcriptome. Therefore, an analysis based on a single transcriptome would consider biological scenarios that the other transcriptome might not, thus biasing the analysis, e.g., it is expected that the methodology would provide nodes linked to secondary metabolism, specifically, CA biosynthesis; however, from one method (Rockhopper.TransPS), such a node was not annotated. Accordingly, it could be inferred that genes associated with CA production are not expressed in the high production condition when indeed they are (See annotations in Figure 9A and reference [35]).

## 4. Discussion

In this work, a parameter optimization of the assembler software Rockhopper2 was undertaken, and the quality of the transcriptome obtained was analyzed using the Transrate Assembly Score (TAS).

The quality of the assembled transcriptomes, using Rockhopper2, is very sensible to the software parameters. Parameter optimization allowed reaching a satisfactory TAS of 0.55092; using the Rockhopper2 default parameters, the TAS was 0.19901. Indeed, this is a reasonable improvement if we consider that Smith and Unna calculated TAS values for 155 assemblies in the range of 0.001 and 0.52. A recent work of Holzer and Marz used different metrics, including TAS, to analyze ten assemblers using different RNA-seq data from diverse sources. The authors reached high TAS values for *Candida albicans* (0.54236) and *Homo sapiens* + *Evola virus* (EBOV) (0.5475) [64]. Values attained by these authors resemble the values reached in the present work for the TAS.

Overall, these results indicate that parameter optimization is needed and highly recommended for better de novo assembly. This work revealed that all parameters in Rockhopper2 define the quality of the assembled transcriptomes in terms of TAS. The parameter “*min count to seed a transcript*” is responsible for the starting of assembling a candidate transcript if at least the specified number of k-mers are present in the reads. Meanwhile, “*min count to extend a transcript*” deals with extending a candidate transcript from a node in the De Bruijn graph if at least the specified number of k-mers in the reads correspond to the subsequent node. Further, a candidate transcript is considered as high quality transcript if it has surpassed the threshold determined by “*minimun transcript length*” and by “*min reads mapping to a transcript”* [5]. In this work, the optimization tended to decrease the parameters *“min reads mapping to a transcript”, “min count to seed a transcript”* and *“min count to extend a transcript”,* which might be caused by the low coverage for the organism under study. Thus, many transcripts present in the sample were not initially assembled since there were not enough reads, and consequently the k-mer frequency was too low to fill the conditions required for the software parameters. By decreasing these parameters, many more contigs can be assembled and more reads can be used for the assembling process, thus increasing quality.

The performance of Trinity has been analyzed elsewhere [19,20,21,22,25,28,65,66,67,68,69,70]. In general, Trinity assembles more contiguous transcripts; it has a good performance at assembling conserved genes across a large set [71]; it also has the ability to detect alternative splice isoforms and does not require parameter tuning [3]. All this in contrast to its long time and high Random Acces Memory (RAM) required for assembly, which make it necessary to have high computational resources. Nowadays, it is available at some platforms e.g., Galaxy [45] or TRUFA [72].

According to a previous report, Trinity produced few chimeras across all single k-mer assemblies of the *Arabidopsis thaliana* transcriptome [70]. Conversely, Yang and Smith reported that Trinity produces large amounts of trans self-chimeras [20]. In this work, six self-chimeras out of 6488 transcripts were found. No additional chimeras were found using Vsearch Quimera Detection [43] and CD-HIT-DUP [44], and three were found using the proposed alternative.

In terms of TAS, the performance of Rockhopper2 and Trinity was quite similar, despite the in-depth parameter optimization in Rockhopper2. Trinity assembles almost double the transcripts compared with Rockhopper2; 94% of the reads align to the assembled transcriptome, which is quite similar to the reference-based approach (97%), yet the mean length is shorter. A high percentage of reads mapping back to the transcriptome assembly is desirable for accurate differential gene expression analysis because more reads mapped back to the assembly will result in increased statistical power for performing these analyses [21].

Redundant contigs represent highly similar sequences corresponding to the same reference. Huang et al. reported that Trinity assemblies contain more redundancy relative to assemblies generated by the genome-guided and TransPS methods. The same authors inferred this is in part due to the fact that RNA-Seq was performed on mRNAs from tissue samples that were pooled from multiple individuals [21]. In this work, the high redundancy in the Trinity assembly might be related to its ability to detect isoforms in eukaryotic genes. Therefore, highly similar transcripts that could codify for the same gene in bacteria are designated as different transcripts because of the underlying software design; yet, it could be related to intra-reads error which might lead to this situation.

Due to its scaffolding process, TransPS increases mean length both in Trinity and Rockhopper2 assemblies. Moreover, the treatment with TransPS allows for retrieving almost double the full-length transcripts assembled than CD-HIT. Therefore, the post-assembly scaffolding process carried out by TransPS is highly recommended considering its benefits in terms of redundancy reduction and full-length transcripts recovered.

Concerning the gene ontology terms annotated, both Trinity.CD-HIT and Trinity.TransPS share a high quantity of GO terms among the DEG, depending on the quantification method used, with 88% and 91% of the terms in common, respectively. The Rockhopper.TransPS transcriptome only reaches a 76% of GO terms in common for both quantification methods (Figure S3). The previous results may be related to the quantification algorithm of Salmon that uses quasi-mapping instead of traditional alignments. Thus, sometimes it could provide superior accuracy by being more robust to errors in the read or genomic variation from the reference sequence [73]. In contrast, RSEM needs a previous alignment using either bowtie [74] or bowtie 2 [58]; this could discard some information depending on the parameter used in the aligner, a topic not explored in this work. The type of data from a bacterial transcriptome where no isoforms are produced and the post-assembly procedures may explain the differences between the quantities of DEG, detected after the different method of transcript quantification. The quantity of genes considered as differentially expressed continues to be greater in the reference-based mode than in de novo; yet, de novo allows for detecting genes that the reference-based does not. This could be related to transcript fragmentation and incomplete and incorrect annotation, whereby we suggest that de novo assembly is beneficial even when a reference genome is available [70].

As a first approach, this study highlights the importance of a deep analysis of the parameters of the assembler software, and the post-assembly methodologies. Consequently, we believe that a deeper evaluation of strategies recommended by other authors, which were successful for improving the quality of assembled transcriptomes from short-reads, are required in prokaryotic transcriptomes, e.g., the use of a deeper sequencing, to merge the results from different assemblies and use a genome-guided de novo assembly. Certainly, the most interesting result will be the different annotations reached depending on the methodology; therefore, the biological conclusions will greatly depend on the selected method.

## 5. Conclusions

Transcriptome analysis has been mostly dedicated to eukaryotic organisms. As far as we know, this is the first work aimed at assessing software for prokaryotic organisms such as Rockhopper2. This study highlights the importance of a parameter optimization and analysis, primarily when a de novo assembly is carried out.

Previous studies reported that Rockhopper2 is superior to Trinity for assembling bacterial transcriptomes [5]; however, our findings contradict this report since the quality of the transcriptome assembled with Trinity surpassed the quality of the transcriptome assembled with Rockhopper2, even after the parameter optimization study; nonetheless, the quality was quite similar.

The analysis cannot be limited to TAS; further methodologies must be used such as completeness and redundancy. Thus, a wider panorama about the quality of the assembled transcriptome is achieved. Similar conclusions were reached by Honaas et al. [22], who concluded that the quality of de novo transcriptome assemblies is best assessed through consideration of a combination of metrics. CD-HIT does not provide proper results when removing redundancy, since various transcripts with duplicates are detected as differentially expressed. For this, a methodology based on homology searching using BLAST, such as TransPS, is suggested.

Finally, appropriate techniques for transcriptome analysis are highly recommended since biological outcomes will rely on the selected method. Thus, different annotations, depending on the method for removal of redundancy and transcript quantification, may be accomplished.

## Figures and Tables

**Table 1:** Comparison of different metrics for the assembled transcriptome through distinctive software.

Quality Metric	Rockhopper2 Default Parameters	Rockhopper2 Optimized Parameters	Trinity Default Parameters
**Number of Sequences**	1129	3459	6488
**Mean Length**	243.13	604.088	483.83
**Number with ORF**	165	1528	1919
**Fragments**	638,046	638,046	638,046
**Mapped**	478,534 (75%)	571,882(89%)	602,400(94%)
**TAS**	0.19001	0.55092	0.58337

**Table 2:** Quality control of the assembled transcriptomes subjected to different strategies for removing redundancy.

Quality Metric	Trinity CD-HIT	Trinity TransPS	Rockhopper TransPS
**Number of Sequences**	6373	3825	2481
**Mean Length**	479.36	743.95	687.89
**Number with ORF**	1861	1758	1323
**Fragments**	638,046	638,046	638,046
**Mapped**	602,657 (94%)	555,748 (87%)	530,315(83%)
**TAS**	0.6122	0.5303	0.5405

## Supplementary Materials

The following are available online at https://www.mdpi.com/2571-5135/8/4/20/s1, Figure S1: Quality control, Figure S2: MA and Volcano plots, Figure S3: Gene ontology terms shared between transcriptomes evaluated, Table S1: Basic statistics of the pre-assembly stages, Table S2: Factorial design for the Rockhopper2 parameters in de novo mode, Table S3: Central composite design 1 for the Rockhopper2 parameters in de novo mode, Table S4: Central composite design 2 for the Rockhopper2 parameters in de novo mode.
