# Supplementary material for: Comparative Analysis of Strategies for De Novo Transcriptome Assembly in Prokaryotes: Streptomyces clavuligerus as a Case Study
Source: High Throughput. 2019 Nov 30;8(4):20. doi: 10.3390/ht8040020 (PMC6970227; doi:10.3390/ht8040020)
Supplement: Supplementary file 1 [file high-throughput-08-00020-s001.pdf]

Supplementary Material

Comparative Analysis of Strategies for De Novo Transcriptome Assembly in Prokaryotes: *Streptomyces clavuligerus* as a case study

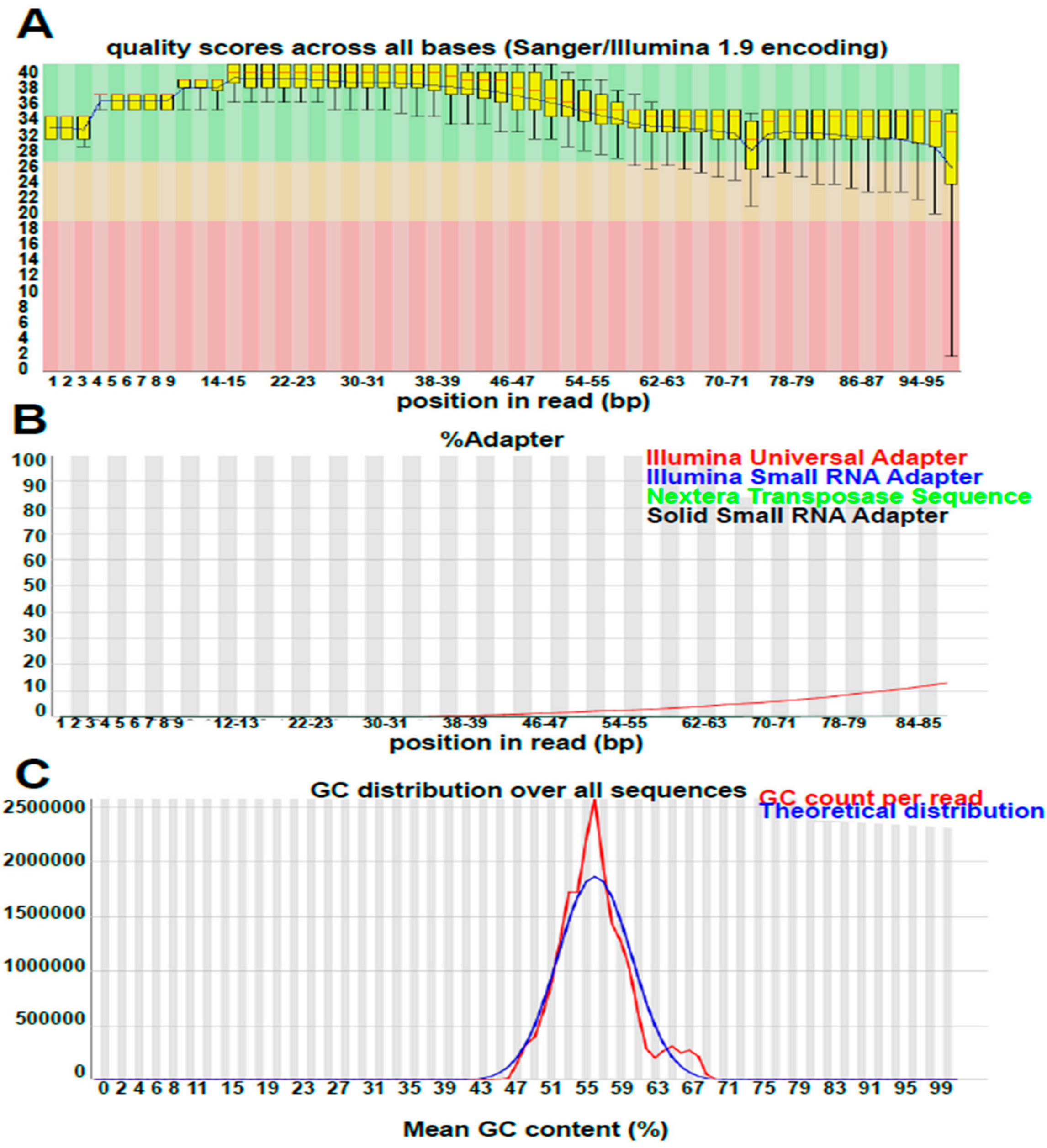

Figure S1. (A) Quality control across all bases **(B)** Adapter content **(C)** GC distribution over all sequences. The plots were generated for the High production condition library. Similar results were obtained for the quality control in the low production condition library.

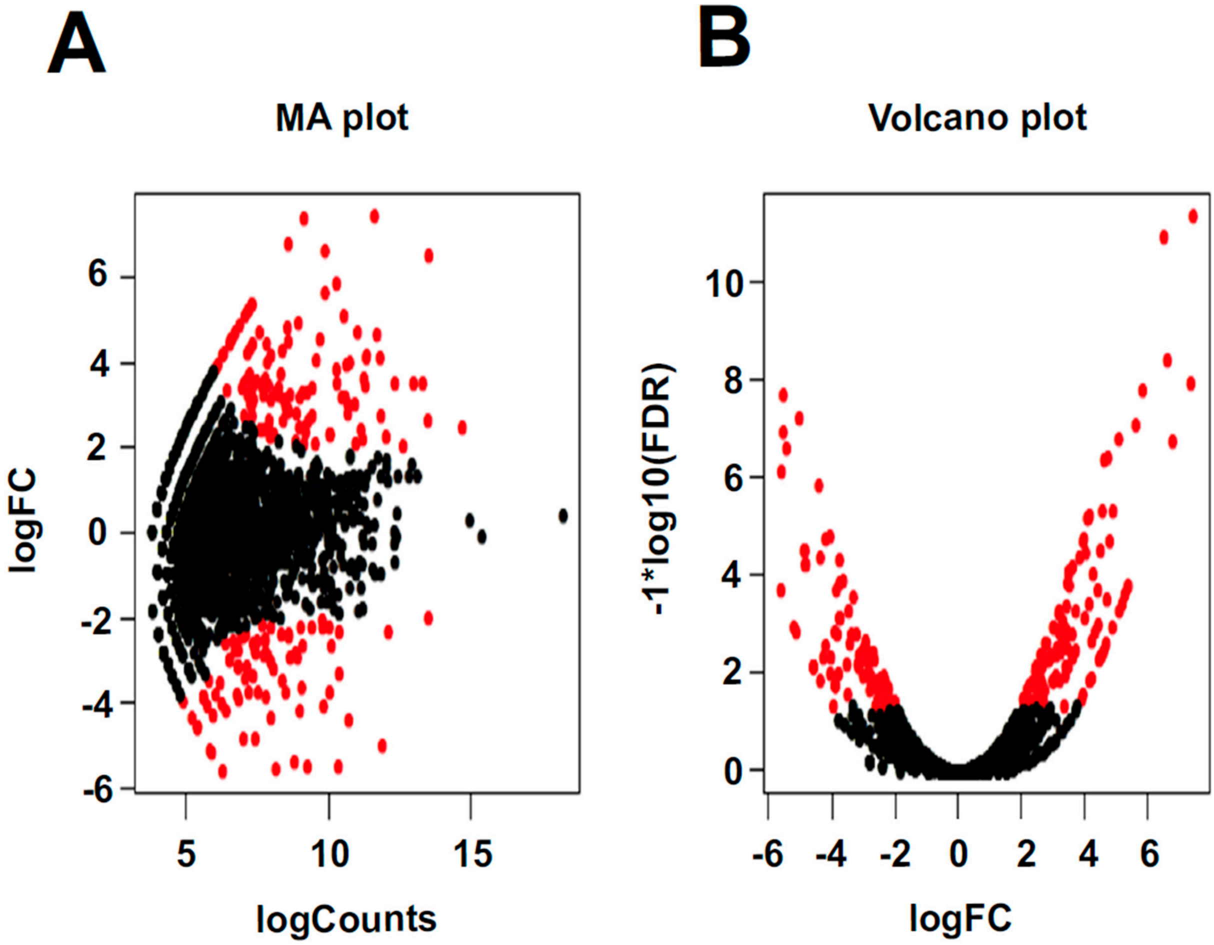

Figure S2. (**A**). MA-plot (Abundance vs fold change) for the Rockhopper.TransPS transcriptome and Salmon as quantification software. (**B**) Volcano plot (Fold chance vs significance) for the Rockhopper.TransPS transcriptome and Salmon as quantification software.

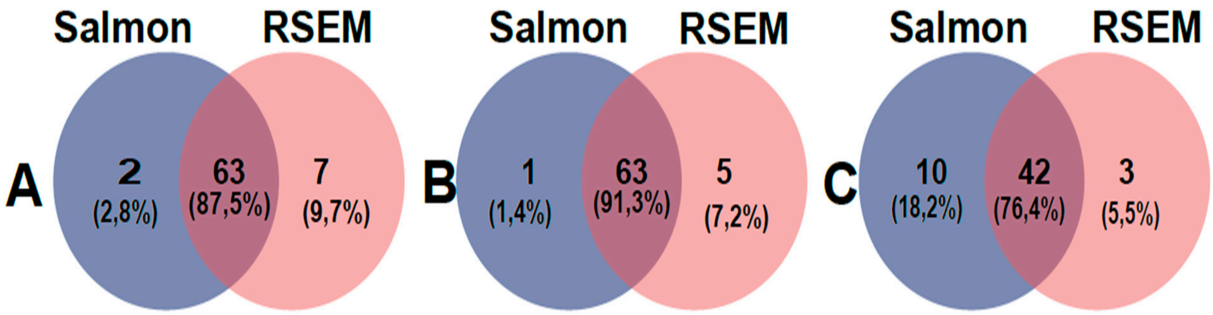

Figure S3. Gene ontology terms shared between transcriptomes evaluated. (**A**) Trinity.CD-HIT (**B**) Trinity.TransPS (**C**). Rockhopper.TransPS.

Table S1. Basic statistics of the pre-assembly stages. rRNA depleted with SortMeRNA. Adapters trimmed with Trimmomatic.

Feature\Condition	High Production	Low Production
**Raw reads**	23615874	20165574
**Length(bp)**	101	101
**Coverage**	557.4	476.0
**Reads after rRNA depletion**	536785	274638
**Percentage of rRNA**	97.73	98.64
**Coverage after rRNA depletion**	12.67	6.48
**Reads after adapter trimming**	454613	187617
**Percentage of adapters**	0.35	0.43
**Coverage after adapter trimming**	10.73	4.43

Table S2. Factorial design for the Rockhopper2 parameters in de novo mode. A: Min reads mapping to a transcript. B: Minimum transcript length. C: Min count to seed a transcript D: Min count to extend a transcript. TAS: TRANSRATE ASSEMBLY SCORE.

Run	A	B	C	D	TAS	Run	A	B	C	D	TAS
**1**	5	10	90	1	0.1389	**42**	20	90	50	5	0.1605
**2**	5	10	50	1	0.1287	**43**	5	10	50	9	0.2426
**3**	35	10	10	5	0.0339	**44**	20	50	90	9	0.1804
**4**	5	90	50	1	0.2746	**45**	35	90	10	9	0.1088
**5**	20	10	10	5	0.046	**46**	20	90	10	1	0.1388
**6**	35	90	50	5	0.1009	**47**	20	10	50	1	0.0644
**7**	20	90	90	5	0.1971	**48**	5	50	50	5	0.2699
**8**	20	50	50	9	0.1246	**49**	35	10	10	9	0.0346
**9**	5	50	10	5	0.1583	**50**	20	10	10	9	0.0521
**10**	5	50	50	9	0.2424	**51**	20	90	50	1	0.162
**11**	20	50	10	1	0.0895	**52**	35	90	50	1	0.1059
**12**	5	90	10	5	0.1962	**53**	5	90	10	9	0.1858
**13**	5	50	50	1	0.2268	**54**	35	90	10	1	0.1053
**14**	5	10	10	5	0.1416	**55**	20	10	90	1	0.0807
**15**	20	10	50	5	0.0817	**56**	35	10	50	5	0.0356
**16**	20	10	90	5	0.1064	**57**	5	50	10	9	0.167
**17**	35	10	90	9	0.0628	**58**	5	50	10	1	0.1557
**18**	35	50	90	5	0.1057	**59**	20	90	10	9	0.1356
**19**	35	50	90	1	0.1049	**60**	35	90	50	9	0.1088
**20**	20	50	50	5	0.1331	**61**	5	90	90	1	0.2372
**21**	35	50	10	9	0.0688	**62**	5	90	10	1	0.2272
**22**	35	10	50	1	0.0366	**63**	20	90	50	9	0.1456
**23**	35	50	10	5	0.0733	**64**	5	50	90	9	0.2271
**24**	5	90	50	9	0.2423	**65**	5	10	10	1	0.0919
**25**	35	10	90	1	0.0584	**66**	35	10	90	5	0.0606
**26**	35	50	90	9	0.1066	**67**	35	50	10	1	0.0718
**27**	20	90	10	5	0.1416	**68**	5	10	90	5	0.2414
**28**	35	10	10	1	0.0345	**69**	5	10	90	9	0.2271
**29**	35	10	50	9	0.0356	**70**	5	90	90	9	0.2272
**30**	35	50	50	5	0.0741	**71**	35	90	10	5	0.101
**31**	20	50	10	9	0.0977	**72**	20	50	50	1	0.1225
**32**	20	50	10	5	0.098	**73**	5	10	50	5	0.2626
**33**	20	50	90	1	0.1575	**74**	5	50	90	5	0.2414

**34**	20	50	90	5	0.1588	**75**	35	90	90	5	0.1353
**35**	35	90	90	1	0.1391	**76**	5	10	10	9	0.1599
**36**	20	10	90	9	0.1227	**77**	5	90	90	5	0.2414
**37**	20	10	50	9	0.0811	**78**	5	50	90	1	0.2164
**38**	35	50	50	1	0.0728	**79**	20	90	90	1	0.1857
**39**	35	50	50	9	0.0688	**80**	5	90	50	5	0.2737
**40**	20	10	10	1	0.0403	**81**	35	90	90	9	0.1384
**41**	20	90	90	9	0.1896						

Table S3. Central composite design 1 for the Rockhopper2 parameters in de novo mode. A: Min reads mapping to a transcript. B: Minimum transcript length. C: Min count to seed a transcript D: Min count to extend a transcript. TAS: TRANSRATE ASSEMBLY SCORE .

RUN	A	B	C	D	TAS
**1**	9	20	160	1	0.1407
**2**	1	20	160	3	0.2002
**3**	5	90	90	2	0.2408
**4**	1	20	160	1	0.1906
**5**	9	20	20	1	0.1029
**6**	9	160	20	1	0.2766
**7**	1	160	20	1	0.3804
**8**	9	20	20	3	0.1069
**9**	5	90	90	2	0.2408
**10**	1	20	20	3	0.3415
**11**	5	90	90	2	0.2409
**12**	9	160	160	1	0.2253
**13**	5	90	2	2	0.2112
**14**	5	90	90	2	0.2415
**15**	5	90	90	2	0.2408
**16**	1	160	160	3	0.1984
**17**	5	90	90	2	0.2407
**18**	0	90	90	2	0.2147
**19**	9	20	160	3	0.1675
**20**	5	90	90	2	0.2414
**21**	5	90	90	2	0.2414
**22**	5	90	90	2	0.2408
**23**	1	160	160	1	0.1977
**24**	5	0	90	2	0.2281
**25**	5	230	90	2	0.2585
**26**	5	90	90	2	0.2409
**27**	9	160	160	3	0.2242
**28**	5	90	90	2	0.2413
**29**	1	20	20	1	0.3657
**30**	5	90	90	4	0.2408
**31**	5	90	90	0	0.2372
**32**	5	90	230	2	0.1743
**33**	1	160	20	3	0.3495
**34**	5	90	90	2	0.2414
**35**	13	90	90	2	0.2019
**36**	9	160	20	3	0.2621

Table S4. Central composite design 2 for the Rockhopper2 parameters in de novo mode. A: Min reads mapping to a transcript. B: Minimum transcript length. C: Min count to seed a transcript. TAS: TRANSRATE ASSEMBLY SCORE .

RUN	A	B	C	TAS
**1**	5	120	2	0.264
**2**	3	200	22	0.3669
**3**	5	120	18	0.3011
**4**	5	280	2	0.3514
**5**	3	200	10	0.3898
**6**	3	335	10	0.3948
**7**	3	65	10	0.2919
**8**	3	200	0	0.3813
**9**	3	200	10	0.3876
**10**	3	200	10	0.3878
**11**	1	120	18	0.3779
**12**	1	280	18	0.3992
**13**	3	200	10	0.3888
**14**	3	200	10	0.3885
**15**	1	120	2	0.0623
**16**	3	200	10	0.3885
**17**	1	280	2	0.4885
**18**	3	200	10	0.3885
**19**	0	200	10	0.4279
**20**	5	280	18	0.3523
**21**	3	200	10	0.3875
**22**	3	200	10	0.3864
**23**	6	200	10	0.3203
**24**	1	300	2	0.4852
